# Altering ApoE in mice expressing human amylin in the pancreas exacerbates brain amyloid pathology and behavior deficits

**DOI:** 10.1002/alz.093337

**Published:** 2025-01-03

**Authors:** Noah S Leibold, Velmurugan Gopal Viswanathan, Laura Radulescu, Deepak Kotiya, Nirmal Verma, Florin Despa

**Affiliations:** ^1^ University of Kentucky, Lexington, KY USA

## Abstract

**Background:**

The APOE ε4 allele is the most prominent genetic predisposition for sporadic Alzheimer’s disease (AD). Amylin, a neuroendocrine hormone co‐secreted with insulin from the pancreas, is increased in blood in AD and readily forms neurotoxic homo‐ and hetero‐oligomers with β‐amyloid in AD. Previously, we showed that intravenously infused ApoE4 in rats expressing human amylin specifically in the pancreas led to increased brain amylin accumulation. Further, we showed increased blood levels of human amylin disrupts β‐amyloid efflux. Therefore, we investigated whether mice humanized for amylin and ApoE demonstrate ApoE isoform‐specific alterations in cerebrovascular amylin deposition and β‐amyloid homeostasis.

**Methods:**

Six‐month‐old male mice humanized for ApoE3 or ApoE4 and amylin (ApoE3HIP and ApoE4HIP) and mice humanized for amylin without ApoE expression (ApoE‐KO‐HIP) were tested for behavior deficits before brain microvessel isolation and amylin quantification by ELISA. β‐amyloid was quantified using the MesoScaleDiscovery platform. GFAP‐amylin colocalization in the brain was quantified using immunohistochemistry (IHC) while double‐immunofluorescence (double‐IF) and STORM imaging were used for amylin‐ApoE colocalization. Immunoprecipitation experiments were conducted to confirm brain amylin‐ApoE binding interactions.

**Results:**

ApoE4HIP mice demonstrated worsened behavioral deficits when compared to E3HIP mice. IHC of ApoE4HIP and ApoE3HIP brains revealed significantly increased deposits of GFAP and amylin in ApoE4HIP mice. Amylin in brain parenchyma, but not capillaries, was higher in ApoE4HIP vs. ApoE3HIP mice (p<0.0001 and p = 0.4455) while ApoE‐KO‐HIP mice demonstrate reduced amylin in both fractions as measured by ELISA. β‐amyloid 40, but not 38 or 42, levels were significantly higher in ApoE4HIP brain and microvessels (p<0.0001 and p = 0.0123) vs. ApoE3HIP. Analysis of double‐immunofluorescence, STORM, and immunoprecipitation experiments is ongoing.

**Conclusions:**

Our data suggest ApoE may function as a transporter of amyloid‐forming amylin in the brain with amylin binding ApoE4 stronger than ApoE3. The increased affinity of amylin for ApoE4 coincides with worsened brain amyloid pathology, disrupted β‐amyloid homeostasis, increased astrogliosis suggesting neurodegenerative insult, and functional impairments due to elevated amylin amyloid burden. These data may implicate the amylin‐ApoE interaction as a mechanism underlying ApoE4‐specific neuropathology. Additional studies to delineate the mechanisms by which amylin and ApoE interact at the BBB interface and alter amyloid deposition are needed.

